# High prevalence of t895 and t9364 spa types of methicillin-resistant *Staphylococcus aureus* in a tertiary-care hospital in Mexico: different lineages of clonal complex 5

**DOI:** 10.1186/s12866-020-01881-w

**Published:** 2020-07-20

**Authors:** C. Negrete-González, E. Turrubiartes-Martínez, O. G. Galicia-Cruz, D. E. Noyola, G. Martínez-Aguilar, L. F. Pérez-González, R. González-Amaro, P. Niño-Moreno

**Affiliations:** 1 Sección de Genómica Médica, Centro de Investigación en Ciencias de la Salud y Biomedicina, Universidad Autónoma de San Luis Potosí, San Luis Potosí, Mexico; 2 Laboratorio de Hematología, Facultad de Ciencias Químicas, Universidad Autónoma de San Luis Potosí, San Luis Potosí, Mexico; 3 Departamento de Farmacología, Facultad de Medicina, Universidad Autónoma de San Luis Potosí, San Luis Potosí, Mexico; 4 Departamento de Microbiología, Facultad de Medicina, Universidad Autónoma de San Luis Potosí, San Luis Potosí, Mexico; 5 Unidad de Investigación Biomédica, Instituto Mexicano del Seguro Social, Durango, Mexico; 6 Hospital Central “Dr. Ignacio Morones Prieto”, San Luis Potosí, Mexico; 7 Sección de Medicina Molecular y Traslacional, Centro de Investigación en Ciencias de la Salud y Biomedicina, Universidad Autónoma de San Luis Potosí, San Luis Potosí, Mexico; 8 Laboratorio de Genética, Facultad de Ciencias Químicas, Universidad Autónoma de San Luis Potosí, San Luis Potosí, Mexico

**Keywords:** Methicillin-resistant *Staphylococcus aureus*, Spa-typing, SCC*mec* type II, Clonal complex 5-ST1011, Spa type t895, Spa type t9364, New York/Japan-Mexican variant clone

## Abstract

**Background:**

*Staphylococcus aureus* is a leading cause of broad-spectrum infections both in the community and within healthcare settings. Methicillin-resistant *Staphylococcus aureus* (MRSA) has become a global public health issue. The aim of this study was to examine the clinical and molecular characteristics of *Staphylococcus aureus* isolates and to define the population structure and distribution of major MRSA clones isolated in a tertiary-care hospital in Mexico.

**Results:**

From April 2017 to April 2018, 191 *Staphylococcus aureus* isolates were collected. The frequency of MRSA was 26.7%; these strains exhibited resistance to clindamycin (84.3%), erythromycin (86.2%), levofloxacin (80.3%), and ciprofloxacin (86.3%). The majority of MRSA strains harbored the SCC*mec* type II (76.4%) and t895 (56.8%) and t9364 (11.7%) were the most common spa types in both hospital-associated MRSA and community-associated MRSA isolates. ST5-MRSA-II-t895 (New York /Japan clone) and ST1011-MRSA-II-t9364 (New York /Japan-Mexican Variant clone) were the most frequently identified clones. Furthermore, different lineages of Clonal Complexes 5 (85.4%) and 8 (8.3%) were predominantly identified in this study.

**Conclusion:**

Our study provides valuable information about the epidemiology of MRSA in a city of the central region of Mexico, and this is the first report on the association between t895 and t9364 spa types and ST5 and ST1011 lineages, respectively. These findings support the importance of permanent surveillance of MRSA aimed to detect the evolutionary changes of the endemic clones and the emergence of new strains.

## Background

*Staphylococcus aureus* (*S. aureus*) is a commensal and a pathogen in humans; approximately 30–50% of the population are transient nasal carriers and 10–20% of individuals are persistently colonized with this organism [1, 2]. Furthermore, colonization of the skin or mucosa with *S. aureus* may increase the risk of invasive infections [3]. In addition, *S. aureus* has been recognized as an extremely versatile pathogen in humans, causing three major syndromes: superficial lesions, such as impetigo and skin wound infections; deep and systemic infections, such as osteomyelitis, endocarditis, pneumonia, and bacteremia; and toxemic infections, such as toxic shock syndrome, scalded skin syndrome, and food poisoning [4].

In 1961, one year after the introduction of methicillin into medical practice, the first methicillin-resistant *Staphylococcus aureus* (MRSA) strain was identified; methicillin resistance is mediated by the Staphylococcal Cassette Chromosome *mec* (SCC*mec*) genetic element [5]. This element includes the *mec* and *ccr* gene complexes, which are flanked by three junkyard regions. SCC*mec* is inserted into a unique site of the bacterial chromosome by the action of Ccr proteins (encoded by the *ccr* gene complex), which induce the specific recombination between the attB sequence at the 3′ end and the attS homologous sequence of SCC*mec* [6]. Variations in the genetic content and structural organization of these elements result in 13 different types and subtypes of SCC*mec* [7–9].

An increasing number of MRSA strains were identified initially in hospital centers (HA-MRSA) and, several years later, cases of community-associated MRSA infections (CA-MRSA) were reported. In this regard, the epidemiology of MRSA infections has changed significantly with the global emergence and expansion of CA-MRSA strains [10].

The most frequently reported MRSA isolates belong to major Clonal Complexes (CC) CC1, CC5, CC8, CC22, CC30, CC45, and CC80 [11–13]. The most representative HA-MRSA clones are ST5-I/EMRSA-3/Cordobes-Chilean and ST5-II/USA100/New York/Japan clones (CC5), ST36-II/USA200 clone (CC30), ST45-II/USA600 clone (CC45), and ST239 III/ Brazilian/Hungarian clone (CC8), while the most representative CA-MRSA are ST1-IV/USA400 (CC1), ST5-IV/Pediatric clone (CC5), ST8-IV/USA300 and USA300-LA variant (CC8), EMRSA-15 clone (CC22), ST30-IV/Southwest Pacific clone (CC30), and ST80-IV/European clone (CC80) [12, 14]. The distribution of these clones varies in different countries and regions of the world; in Mexico, ST5-II/New York Japan and USA300 clones have been described [15, 16].

According to the World Health Organization (WHO), global surveillance of MRSA is essential for the identification of international transmission routes and the subsequent development of effective prevention and control strategies of this pathogen [17]. For this purpose, molecular typing methods are a valuable tool for the successful characterization of *S. aureus* isolates. In this regard, Next Generation Sequencing (NGS) has been used to identify *S. aureus* CCs and is considered the best laboratory technique for identification of DNA diversity in any organism. However, this methodology remains technically demanding and requires robust software to analyze the results [12]. Traditional typing methods include Multiple Locus Sequence Typing (MLST), Pulsed-Field Gel Electrophoresis (PGFE), and spa-typing. MLST is a great tool for evolutionary investigations and strain identification and is based on the allelic profile of the seven housekeeping genes. PFGE is based on the digestion of DNA with restriction endonucleases and the detection of the banding patterns. Although these two methods show a high discriminatory power, they are laborious and require intra-laboratory standardization protocols [12]. On the other hand, spa-typing is based on the detection of sequence variation in repeats at the X region of the staphylococcal protein A *spa* gene. This typing technique exhibits high discriminatory power, has a standardized nomenclature, is cost-effective, and shows an excellent reproducibility. Spa-typing can be used for the investigation of hospital outbreaks and to analyze the evolution of *S. aureus* [18]. However, this methodology has some limitations, mainly in regions where a particular clone or a small number of clones are endemic [11, 19].

The aim of this study was to estimate the prevalence of MRSA and to analyze the molecular characteristics, and antibiotic resistance profiles of CA- and HA-MRSA genotypes in San Luis Potosi, a large city (approximately 1.1 million inhabitants) in the center of Mexico.

## Results

### Sample collection

*S. aureus* strains were obtained from one hundred ninety-one patients from the emergency department (*n* = 62), surgery (*n* = 47), intensive care unit (*n* = 31), internal medicine (*n* = 35), gynecology (n = 6), burn unit (*n* = 2), and outpatient service (*n* = 8); patients in whom samples were obtained in the outpatient service were subsequently admitted to the hospital. The clinical specimens were obtained from infections in skin and soft tissues (*n* = 79), respiratory tract (*n* = 53), blood (*n* = 36), bone and joints (*n* = 20), and cerebrospinal fluid (*n* = 3). Seventy-seven percent (147 out of 191) of strains were considered as HA and 23 % (44 out of 191) were classified as CA.

One hundred fourteen patients were male and seventy-seven were female. Forty isolates were identified in children, and 151 in adults. The median age was 44 years. The mean length of hospital stay was 18.4 ± 19.5 days (range 1–105 days). Table 1 shows comorbidities, surgical procedures, and history of hospital admission in the last two years before infection of participants in the study. The majority of patients (84.4%) were discharged due to clinical improvement, 2% of patients were transferred to another hospital, 1.6% of patients requested voluntary discharge, and 11.5% of patients infected with *S. aureus* died.

**Table 1:** Clinical and demographic characteristics of the patients with *S. aureus* infection included in the study

	***N*** = 191	(%)
**Sex**		
Male	114	59.7
Female	77	40.3
**Age (years)**		
Infants 0–1	12	6.2
Children 2–10	13	6.8
Adolescents 11–17	15	7.8
Young adults 18–35	59	30.9
Adults 36–60	62	32.5
Seniors > 60	30	15.7
**Length of stay (days)**		
Mean	18.45	
SD	19.52	
Range	1–105	
**Underlying disease**		
Diabetes mellitus	51	26.7
Hypertension	45	23.6
Renal disease	21	11
Neoplasms	10	5.2
**Surgical procedures**	84	44
**Prior hospitalization**	135	70.7
**Hospital discharge**		
Clinical improvement	162	84.4
Death	22	11.5
Transfer	4	2.1
Voluntary discharge	3	1.6

Ten (45.5%) of the 22 patients who died had an MRSA infection compared to 41 (24.3%) of the 169 patients who survived (*P* = 0.034). Patients who died were also older (mean 46.3 years) than those who survived (mean 35.2 years; *P* = 0.034). In contrast, there were no significant differences in the prevalence of underlying conditions (such as diabetes, malignancy, or renal disease) between patients with a fatal outcome and those who did not die (Additional file 1: Table S1).

### Identification of MRSA strains

**Fig. 1:**
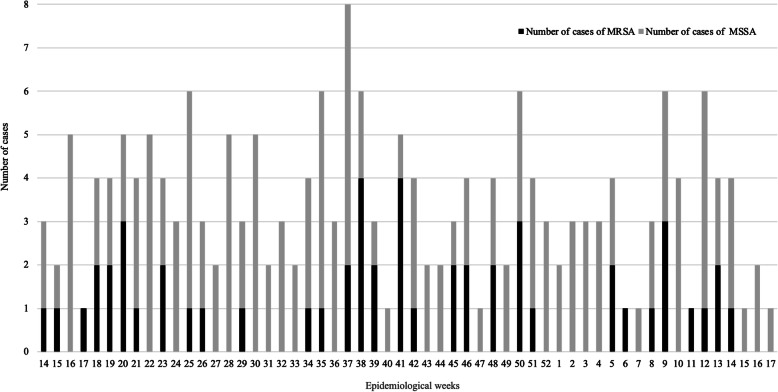
Number of cases of *S. aureus* infection in each epidemiological week. Black bars correspond to MRSA isolates and grey bars to MSSA strains

The *mecA* gene was detected in 51 out of 191 isolates (26.7%), and 45 of them showed resistance to oxacillin and were positive on cefoxitin-based screening. The study was carried out between epidemiological week (as defined by WHO) 14, 2017 and epidemiological week 17, 2018. The weekly number of *S. aureus* infections varied between 1 and 8 cases. As shown in Fig. 1, the largest number of cases was observed at week 37 (eight, two of them MRSA), whereas in weeks 25, 35, 38, 50, 9 and 12, six cases were identified. Moreover, the highest weekly number of MRSA cases was 4, in weeks 38 and 41, followed by weeks 20, 50, and 9 with 3 cases. Two MRSA cases were identified in weeks 18, 19, 23, 37, 39, 45, 46, 48, 5, and 13, whereas a single case was detected in weeks 14, 15, 17, 21, 25, 26, 29, 34, 35, 42, 51, 6, 8, 11, 12, and 14. No MRSA isolates were observed during weeks 16, 22, 24, 27, 28, 30–33, 36, 40, 43, 44, 47, 49, 52, 1–4, 7, 10, 15–17. Three different periods of MRSA detection were identified during the study. The first period occurred between weeks 14 and 29 (2017), the second between weeks 34 and 51(2017), and the third between weeks 5 and 14 (2018) Fig. 1.

### Antimicrobial susceptibility

The antibiotic resistance pattern differed significantly between MRSA and MSSA isolates (*P* < 0.001 in most cases). Thus, most MRSA strains showed resistance to clindamycin (84.3%), erythromycin (86.2%), levofloxacin (80.3%), and ciprofloxacin (86.3%), with low resistance to gentamicin (13.7%) and rifampin (9.8%). In contrast, MSSA strains showed minimal resistance to clindamycin (7.1%), erythromycin (9.3%), ciprofloxacin (3.5%), levofloxacin (1.4%), and gentamicin (1.4%). None of MRSA or MSSA strains were resistant to vancomycin, linezolid, tigecycline, trimethoprim/sulfamethoxazole, and tetracycline (Table 2). Additional file 2: Table S2, shows the minimum inhibitory concentration for each antibiotic.

**Table 2:** Antibiotic resistant pattern of MSSA and MRSA isolates

	MSSA ***N*** = 140	MRSA ***N*** = 51	P
Antibiotic	(n / %)	(n / %)	
Benzylpenicillin	109 (77.8)^a^	51 (100)	
Clindamycin	10 (7.1)	43 (84.3)	< 0.001
Erythromycin	13 (9.3)	44 (86.2)	< 0.001
Levofloxacin	2 (1.4)	41 (80.3)	< 0.001
Ciprofloxacin	5 (3.5)	44 (86.3)	< 0.001
Moxifloxacin	0 (0)	18 (35.2)	< 0.001
Rifampin	0 (0)	5 (9.8)	0.001
Gentamicin	2 (1.4)	7 (13.7)	0.002
Oxacillin	0 (0)	45 (88.2)	< 0.001
Vancomycin	0 (0)	0 (0)	NA
Tetracyclin	0 (0)	0 (0)	NA
Linezolid	0 (0)	0 (0)	NA
Tigecycline	0 (0)	0 (0)	NA
Trimethoprim/sulfametoxazole	0 (0)	0 (0)	NA

^a^The penicillinase test was not performed in the 31 benzylpenicillin susceptible MSSA isolates

### SCC*mec* typing

Thirty-nine MRSA strains were classified as SCC*mec* type II (four CA-MRSA, and thirty-five HA-MRSA) and the SCC*mec* subtype IIb was identified in four strains (HA-MRSA). Two isolates harbored SCC*mec* type IVc/E and SCC*mec* type IVa was identified in one isolate (one CA-MRSA and two HA-MRSA). In five isolates it was not possible to identify the SCC*mec* types.

#### Spa-typing

MRSA isolates were classified in 11 different spa types, including t895 (*n* = 29, 56.8%), t9364 (*n* = 6, 11.7%), t008 (n = 2, 3.9%), t003 (*n* = 3, 5.8%), t4229, t002, t012, t040, t304, t111, and t509 (*n* = 1). The spa type t895 was the most common spa type among HA-MRSA and CA-MRSA isolates. In one strain, we identified a spa type not previously reported (spa type unknown). In three isolates, the PCR employed by us did not amplify the *spa* gene.

### Dendrogram of MRSA strains

**Fig. 2:**
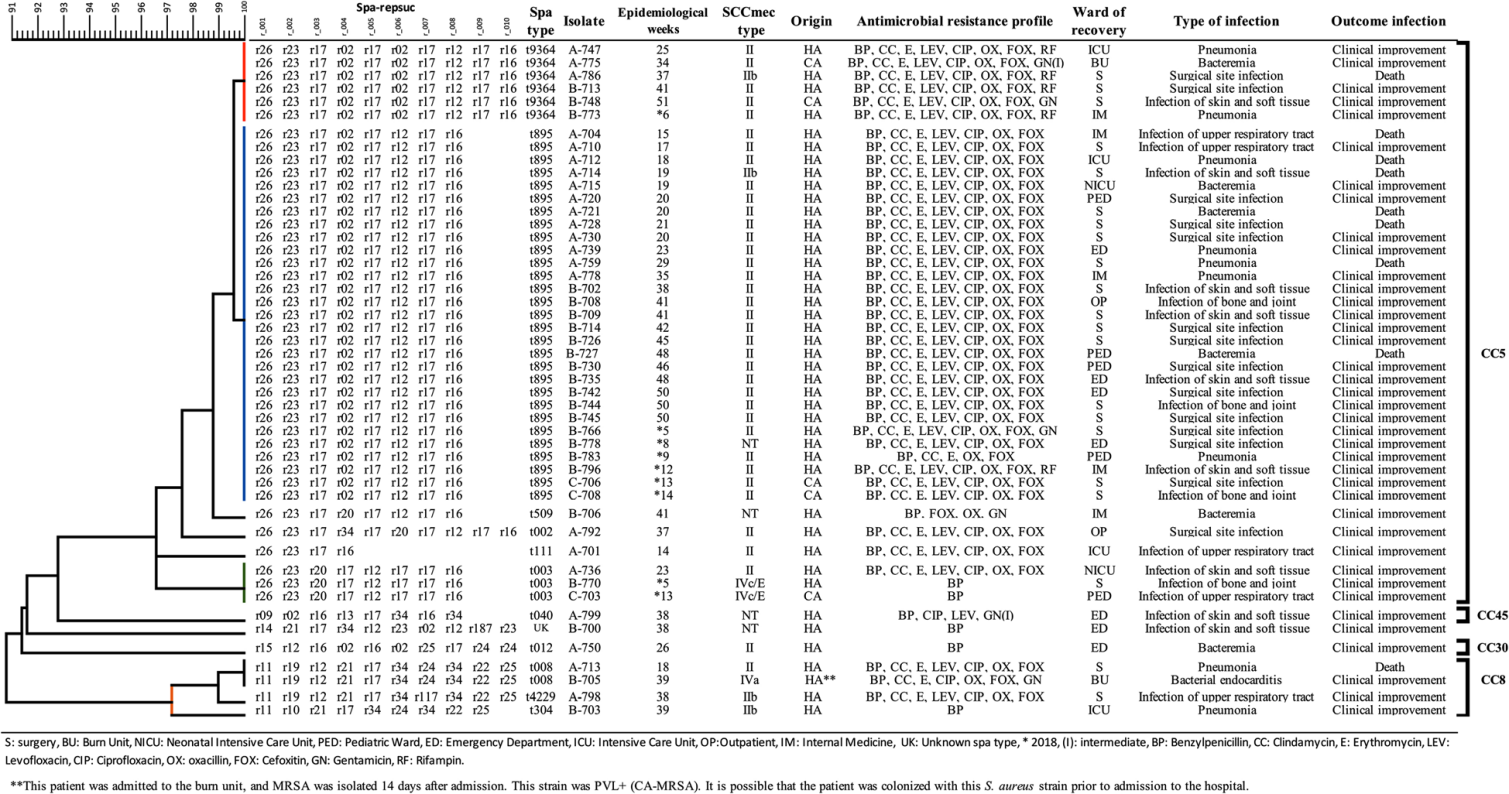
Dendrogram of MRSA strains. Dendrogram constructed using the unweighted pair group method with arithmetic average (UPGMA) based on pairwise similarity values of spa types from 48 characterized MRSA strains. The scale corresponds to the percent of similarity. Blue branch corresponds to cluster 1, red branch to cluster 2, green branch to cluster 3, and orange branch to cluster 4

A dendrogram was constructed to analyze the relation among *S. aureus* strains based on their spa type (Fig. 2). The spa type t895 (cluster 1) was identified in twenty-nine isolates in patients from the surgery ward (*n* = 15), emergency department (*n* = 4), internal medicine (*n* = 3), intensive care unit (*n* = 1), and outpatient service (*n* = 1). In children, t895 was identified in the pediatric ward (*n* = 4) and neonatal intensive care unit (n = 1). Twenty-seven isolates harbored the SCC*mec* type II, one isolate harbored SCC*mec* type IIb, and in one strain the SCC*mec* type was not identified. Twenty-seven isolates of this cluster showed resistance to beta-lactams, fluoroquinolones (levofloxacin and ciprofloxacin), clindamycin, and erythromycin. The B-796 strain was rifampin-resistant, and the B-766 strain was gentamicin-resistant. The origin of MRSA strains in this cluster was predominantly HA-MRSA (*n* = 27), and only two cases were CA-MRSA (C-706 and C-708); the latter isolates were identified in the surgery ward, in weeks 13 and 14. In this cluster seven patients died.

The cluster 2 (t9364) included six isolates, three from the surgery ward and one from the intensive care unit, the burn unit and the internal medicine ward. Five isolates in this cluster harbored the SCC*mec* type II, and one the SCC*mec* type IIb; strains from both SCC types showed resistance to fluoroquinolones, clindamycin, and erythromycin. Four of these strains (A-747, A-786, B-713, and B-773) were resistant to rifampin, and another (B-748) to gentamicin. The percent of similarity between spa types t895 and t9364 was 99.5%. The A-792 (t002) and B-706 (t509) isolates showed more than 98% of similarity with t985 and t9364, and A-701 (t111) showed 97.6% of similarity to spa types t895, t9364 and t003.

The cluster 3 (t003) included three isolates, two of them were detected in children (A-736 and C-703), and one (B-770) from a patient in the surgery ward. In this group, two isolates harbored SCC*mec* type IVc/E and were resistant to beta-lactams. In the other isolate we identified SCC*mec* type II.

The spa type t040 had a 92.7% similarity with the spa types t895, t9364, t003 and t002, and had 91.5% similarity with an unknown spa type that was identified in B-700. This strain was only resistant to beta-lactams.

The cluster 4 had 90.8% similarity with the previously mentioned spa types. This cluster included four isolates with t008, t4229 and t304 spa types. Two patients from the surgery and burn wards were infected with HA-MRSA-t008, A-713 strain was isolated in the eighteen week and harbored the SCC*mec* type II, and B-705 was isolated in the thirty-nine week and harbored the SCC*mec* type IVa.

### Multilocus sequence typing

Spa types t895 and t9364, the major spa types identified in this study, have not previously been associated to sequence types (ST). In order to analyze this, we selected six isolates, and these were identified as ST1011 (*n* = 4) and ST5 (*n* = 2). The association analysis of spa types of clusters 3 to 4 with ST was performed in the Spa server.

## Discussion

In the present study, we have assessed the epidemiological characteristics of *S. aureus* isolates during one year of intra-hospital surveillance and we analyzed the molecular characteristics of MRSA strains. The most frequent *S. aureus* infections were those affecting the skin and soft tissues (*n* = 48, 25.1%) and bacteremia (*n* = 31, 17%). In contrast, the most frequent type of infection caused by MRSA isolates was surgical site infection (*n* = 14, 27%).

The mortality associated with staphylococcal infections in our study (11.5%) was lower than that previously reported (approximately 15 years ago) in Mexico (50%) [20]. MRSA infections were detected more frequently in fatal cases than in patients who survived. Study participants who died were also older than those who survived. Of note, while the presence of underlying diseases, history of surgical procedures, and health-care exposure have previously been reported to be associated with fatal infections [21, 22], we did not find significant differences for these conditions between patients who died and those who survived.

The recent epidemiology of *S. aureus* has focused on the increase and spread of MRSA strains in the healthcare setting and the community. In Denmark and Scandinavian countries the prevalence of MRSA is less than 1%. In contrast, in the east and southeast of Europe, the prevalence of MRSA is greater than 30% [22]. Peru has the highest reported prevalence in Latin America (80%) [17]. In Mexico, there are a limited number of studies about MRSA and the available information shows an increase in the prevalence of MRSA ranging from 7 to 53% between 1989 and 2017 [15, 23–25]. In our study, the prevalence of MRSA identified by molecular methods was higher than the prevalence identified by the oxacillin resistance phenotype, which has been the most used method in our country [17, 25, 26]. The 26.7% prevalence registered in our study is higher than that reported, between January and June 2018, in 47 hospitals in 20 states of Mexico (21.4%) [26], a study that did not include information from the state of San Luis Potosi. In a previous study, performed between 2005 and 2006 in six Mexican hospitals, the prevalence of MRSA ranged from 1 to 43%. The highest prevalence was recorded at the Hospital Central Dr. Ignacio Morones Prieto (HCIMP) [27]. In 11 years, the prevalence of MRSA decreased to 26.7% in this hospital. This fact can be explained by the infection control actions implemented. A study that highlights the importance of intra-hospital surveillance of MRSA was carried out between January 1997 and May 2003 at the Pediatric Hospital of the Centro Medico Nacional-Siglo XXI (Mexico City). At this hospital, the annual frequency of methicillin resistance ranged from 17 to 23% between 1997 and 2001, and dramatically decreased in 2002 (4%) and 2003 (0%), due to the intervention of the infection control committee at the end of 2001 [28].

Until February 2020 the Spa server has recorded 19,255 different spa types [29]. According to a literature review, in the last decade, the spa types t032/t008/t002 are the most prevalent in Europe, t037/t002 in Asia, t008/t002/t242 in America, t037/t084/t064 in Africa, and t020 in Australia [5]. Interestingly, in our study the prevalence of the spa types commonly described in America was lower than expected, and we mainly detected the spa types t895 and t9364.

Compared to other spa types, t895 has a low frequency (0.01%); however, in the last two years its detection has increased. Between 2017 and 2019, eight strains were reported in USA and another in Germany, according to the Spa Server [29]. Although data is scarce, previous reports have associated the t895 spa type with CC5 [30]. In this regard, our data suggest an apparent association between t895 spa type and the ST5 lineage of CC5. In 97% of the MRSA-t895 isolates, the SCC*mec* type II cassette was identified; these molecular characteristics correspond to the New York / Japan/ USA100 clone [15]. However, a limitation of this study is that the sequence types (MLST) of several of the t895 strains were not determined, which precluded a proper statistical analysis. Of note, SCC*mec* type I, type II, and type IV have been reported previously in MRSA-t895 strains [31].

The identification of t895 as the predominant spa type in our study is of relevance, since this may have clinical and epidemiological implications. Of interest, characterization of 21 MRSA strains isolated in Estado de Mexico (Mexico) in 2013 also showed t895 to be the predominant spa type, accounting for 76.2% of isolates [31]. In a study conducted in the United States, t895 spa type was predictive for the weak-biofilm producing phenotype, compared to t008 spa type, which was identified as a predictor of the strong-biofilm producing phenotype [30].

The spa type t9364 was registered in 2011 and corresponded to a strain detected in Mexico, in a region outside of the state of San Luis Potosi [29]. In this regard, our data describe, for the first time, the association between the t9364 spa type and the ST1011 sequence type. Sequence type ST1011 was registered in the MLST database in 2006; the first report of this ST included four clinical MRSA isolates which differed from the sequence type ST5 by the replacement of a nucleotide in the *arcC* gene. Three of these MRSA ST1011 isolates were identified at HCIMP and one at General Hospital of Durango [27]. Between 2008 and 2017, 14 isolates have been reported with the sequence type ST1011 and the SCC*mec* type II [15, 16]; all of these isolates have been identified in Mexico. In 2017, ST1011-II was classified as the New York / Japan clone because of its similarity to ST5-II [15], and in a subsequent phylogenetic analysis of CC5, it was observed that the clones identified in Mexico were grouped in a subclade that was subdivided into two subclades: ST5-II and ST1011-II. This suggests that ST1011-II is not a New York/ Japan clone, but it may be a variant of it that originated in the late 1990s, the period when the CC30 was replaced in Mexico [16]. In all, available data suggests that ST1011-II-t9364 may be a Mexican variant of the New York / Japan clone which has increased in prevalence in the last 11 years; however, more studies are required to determine the differences with ST5-II-t895 [16].

Other spa types identified with lower frequency in this study corresponded to t111, t509, t003, t012, t040, t4229, and t304. The spa type t003 has been related to ST225 and ST270 sequence types, which are part of CC5 and includes the Rhine Hesse, EMRSA-3 and New York/Japan clones [32]. In addition, the spa types t012 and t040 have been identified in strains belonging to CC30 and CC45, respectively. Furthermore, the spa types t4229 and t304 have been associated with ST8, ST247, ST250, and ST254 sequence types, which belong to CC8 and include the USA300, ORSA IV and Archaic/Iberian clones [33].

Diverse lineages of CC5 were predominant in our study. These strains are characterized by bearing the SCC*mec* I, II, and IV type cassettes with subtypes IVa, IVc/E, and IVg. In this regard, different studies have shown that most strains of this CC are multi-resistant, mainly to fluoroquinolones, aminoglycosides, macrolides, lincosamides, and streptogramins (as we detected in the isolates of our study), except for those that carry the IVc/E cassette that only show resistance to beta-lactams [15]. Moreover, to determine the relationship between MRSA strains, we classified them into clusters and analyzed their clinical and molecular characteristics. In this analysis, clusters 1 and 2 were distributed in all areas of the hospital within the three periods described previously; in this regard, it is possible that these three periods could be due to different introductions of clones into the hospital or be a consequence of intra-hospital transmission [34]. Although these two possibilities are plausible, the last one could have resulted from transfer of patients between different hospital wards during their stay. Moreover, all strains grouped in these two clusters were multi-resistant, and the highest number of deaths was recorded in cluster 1. Furthermore, most strains in cluster 3 were only resistant to beta-lactams and the methicillin resistance phenotype was not identified. Finally, three out of four isolates in cluster 4 were identified in weeks 38 and 39, the epidemiological weeks with the highest number of *S. aureus* infections. The use of efficient and accurate epidemiological typing methods is a requisite for monitoring the spread of epidemic clones within and between hospitals. In this case, spa-typing was a good tool for differentiate into CC5 lineages, because t895 and t9364 are not widespread spa types [19]. It is worth mentioning that if t895 and t9364 clones become endemic and spread to multiple regions of Mexico, the discriminating power of spa-typing to analyze nosocomial transmission would decrease. To overcome this limitation, recent studies suggest the use of a combination of different typing techniques to increase the ability to discriminate isolates [35].

In our study, all *S. aureus* strains were susceptible to tetracycline, doxycycline and minocycline [36], and trimethoprim-sulfamethoxazole. This observation is of relevance, since these are alternatives for ambulatory treatment of MRSA infections, such as skin and soft tissue infections [37]. In contrast, tetracycline resistance was reported in 6% of MSSA strains and 17% of MRSA strains collected globally between 1997 and 2016 [38]. Resistance to this antibiotic in *S. aureus* is encoded by the *tet*K and *tet*M genes [39], mainly detected in SCC*mec* III, IV, and V MRSA strains [9, 40, 41]. The majority of MRSA strains in our study had SCC*mec* type II, and this might explain, in part, the absence of tetracycline resistance.

## Conclusions

Our data indicate that the most prevalent clones in all areas of our hospital were ST5-MRSA-II-t895 (New York /Japan clone) and ST1011-MRSA-II-t9364 (New York/Japan-Mexican Variant clone), which belong to CC5. In the HCIMP, the dominance of two CC5 lineages is evident; however, MRSA isolates with molecular characteristics consistent with Irish (weeks 18, 38 and 39), USA300 (week 39) and Pediatric (week 13) clones, that are considered epidemic MRSA clones, were identified. We consider that this study further supports continuous molecular monitoring of *S. aureus* infections as a valuable tool for epidemiological surveillance of MRSA since it allows the evaluation of evolutionary changes of endemic clones and the introduction of emerging clones that can cause hospital outbreaks. In addition, subsequent studies that assess the correlation between the phenotype and the MRSA genotype are required, as well as characterization of additional features of these clusters, including virulence factors and resistance genes.

## Methods

### Sample collection

This cross-sectional study was conducted at HCIMP in San Luis Potosi, Mexico, after approval by the Research Committee [COFEPRIS 14 CI 24028083] and the Research Ethics Committee of the HCIMP [CONBIOETICA-24-CEI-001-20,160,427]. The registration number was 29–17. Informed consent was obtained from all participants or legal guardians.

The city of San Luis Potosi is located in central Mexico and is the capital of the state of San Luis Potosi. HCIMP provides medical services to mid- and low-income populations from all over the state; it has 250 beds and 32 beds in the intensive care unit (ICU).

From April 2017 to April 2018, a total of 191 non-repeated *S. aureus* isolates were obtained from different patients in all hospital wards. These isolates were identified by using the Vitek 2C (bioMérieux) system and confirmed by PCR amplification of the *nuc* gene.

Demographic and clinical data, including sex, age, date of hospitalization, type of infection, date of isolation, underlying disease and outcome of infection were collected from medical records. Patients were classified in groups according to their age, as follows: infants (0 to 1-year-old), children (2 to 10 years old), adolescents (11 to 17 years old), young adults (18 to 35 years old), adults (36 to 60 years old), and seniors (more than 60 years old). An infection was considered as CA when symptoms presented < 48 h of a patient’s hospital admission, in the absence of previous healthcare exposure, whereas an infection was considered as HA when occurred 48 h after patient admission or when it was associated with the following risk factors: hospitalization in an acute care unit for at least 48 h in the last year, chemotherapy administration, hemodialysis, wound care, enteric nutrition or specialized nursing care 30 days before the infection [15, 42, 43].

### Antimicrobial susceptibility

Antimicrobial susceptibility testing was performed using Vitek 2C (bioMérieux) and results were interpreted using the Clinical and Laboratory Standards Institute guidelines. Antibiotics tested included benzylpenicillin, clindamycin, erythromycin, levofloxacin, ciprofloxacin, moxifloxacin, rifampin, gentamicin, vancomycin, tetracycline, linezolid, oxacillin, and cefoxitin test [36].

### DNA extraction

Three colonies of an overnight culture were suspended in 100 μL of DNase free water and incubated at 94 °C for 5 min and − 70 °C for additional 5 min. Then, tubes were centrifuged at 13,000 rpm for 5 min and the supernatant was used as DNA template.

### *nuc* and *mecA* identification

All *S. aureus* strains were screened by targeting the *nuc* and *mecA* genes by multiplex PCR (Table 3) [44, 45]. PCR reactions were performed in a 25 μL volume containing 1x of Buffer (200 mM Tris-HCl pH 8.4, 500 mM KCl), 4 mM of MgCl_2_, 10 pmol of each primer, 200 μM of each dNTP’s, 1 U of Taq DNA polymerase and bacterial genomic DNA. The PCR conditions were maintained at 95 °C for 5 min for initial denaturation followed by 30 cycles of 94 °C for 30 s, 60 °C for 30 s, and 72 °C for 30 s. Then, 20 μL aliquots of each sample were subjected to electrophoresis on 2% agarose gel.

**Table 3:** PCR primers used in this study

Gene	Primer	Primer sequence 5′➔3′	Reference
*nuc*	nuc-F	GCGATTGATGGTGATACGGTT	44
	nuc-R	AGCCAAGCCTTGACGAACTAAAGC	
*mecA*	mecA147-F	GTGAAGATATACCAAGTGATT	45
	mecA147-R	ATGCGCTATAGATTGAAAGGAT	
*spa*	1095-F	AGACGATCCTTCGGTGAGC	48
	1517-R	GCTTTTGCAATGTCATTTACTG	
SCC*mec*	Type II-F	CGTTGAAGATGATGAAGCG	46, 47
	Type II-R	CGAAATCAATGGTTAATGGACC	
	Type-IIb-F	TAGCTTATGGTGCTTATGCG	
	Type-IIb-R	GTGCATGATTTCATTTGTGGC	
	Type-IVa-F	GCCTTATTCGAAGAAACCG	
	Type-IVa-R	CTACTCTTCTGAAAAGCGTCG	
	Type IVE-F	CAGATTCATCATTTCAAAGGC	
	Type IVE-R	AACAACTATTAGATAATTTCCG	
	Type IVc-F	CCTGAATCTAAAGAGATACACCG	
	Type IVc-R	GGTTATTTTCATAGTGAATCGC	
*arcC*	arcC-F	TTG ATT CAC CAG CGC GTA TTG TC	50
	arcC-R	AGG TAT CTG CTT CAA TCA GCG	
*aro*	aro-F	ATC GGA AAT CCT ATT TCA CAT TC	
	aro-R	GGT GTT GTA TTA ATA ACG ATA TC	
*glp*	glp-F	CTA GGA ACT GCA ATC TTA ATC C	
	glp-R	TGG TAA AAT CGC ATG TCC AAT TC	
*gmk*	gmk-F	ATC GTT TTA TCG GGA CCA TC	
	gmk-R	TCATTAACTACAACGTAATCGTA	
*pta*	pta-F	GTTAAAATCGTATTACCTGAAGG	
	pta-R	GACCCTTTTGTTGAAAAGCTTAA	
*tpi*	tpi-F	TCGTTCATTCTGAACGTCGTGAA	
	tpi-R	TTTGCACCTTCTAACAATTGTAC	
*yqi*	yqi-F	CAGCATACAGGACACCTATTGGC	
	yqi-R	CGTTGAGGAATCGATACTGGAAC	

### SCC*mec* typing

Identification of SCC*mec* types was performed by multiplex PCR using the genomic DNA from each MRSA isolate, according to a previously described method and primers (Table 3) [46, 47]. DNA amplification was carried out with a 2 min denaturation step at 94 °C, followed by 30 cycles of 60 s at 94 °C for denaturation, 60 s at 55 °C for annealing, and 60 s at 72 °C for extension, and then 5 min at 72 °C for final extension. Then, 20 μL aliquots of each sample were subjected to electrophoresis on 2% agarose gel.

### Spa-typing

The X region of the *spa* gene of each MRSA isolate was amplified by PCR with the primers 1095F and 1517R, as described previously (Table 3) [48]. The amplified products were sequenced, and the results were analyzed using the Ridom Staph Type software version 1.4 (Ridom, GmbH, Wurzburg, Germany [http://spa.ridom.de/index.shtml]) to determine the repeat profile and the spa type of each isolate [29, 49].

### Dendrogram of MRSA strains

Dendrogram was constructed based on spa types data using a temporary BioNumerics evaluation license from Applied Maths (version 7.6, bioMérieux).

### Multilocus sequence typing

MLST was performed on six MRSA strains of the spa types t9364 (*n* = 4) and t895 (*n* = 2). Seven housekeeping genes (*arcC, aroE, glpF, gmk, pta, tpi,* and *yqiL*) of *S. aureus* were used for MLST typing (Table 3). PCRs were carried out in 50 μl reaction volumes containing 10 ng of chromosomal DNA, 10 pmol of each primer, 1 U of Taq DNA polymerase, 5 μl of 10x PCR buffer, and 200 μM each of dNTPs. PCR was performed with an initial denaturation at 95 °C for 5 min, followed by 37 cycles of denaturation at 95 °C for 30 s, annealing at 55 °C for 30 s, extension at 72 °C for 30 s, followed by a final extension step of 72 °C for 5 min [50]. After amplification, the PCR products were purified and sequenced by dideoxynucleotides method (3500 Genetic Analyzer, Applied Biosystems). The consensus sequences were assembled, and the allelic profile was matched using the MLST database (https://pubmlst.org/saureus/).

### Statistical analysis

Comparisons between groups was carried using Fisher’s exact test or the chi-squared test (for categorical variables) and Student’s t test of Mann-Whitney U test (for continuous variables) using Statistical Package for Social Sciences software for Mac OS, version 25.0 (SPSS, IBM, Inc., Chicago, IL, USA). *P* value < 0.05 were considered statistically significant.

## Supplementary information

**Additional file 1: Table S1.** Demographic and clinical characteristics of patients with *Staphylococcus aureus* infections who died or survived. Table S1 shows the demographic and clinical characteristics of patients with *Staphylococcus aureus* infections who died or survived.

**Additional file 2: Table S2.** Minimum Inhibitory Concentration (μg/mL) data for the MRSA strains. Table S2 shows the MIC for each antibiotic for the MRSA strains.

## Data Availability

All data generated or analyzed during this study are included in this published article [and its supplementary information files].

## Abbreviations

*S. aureus*: *Staphylococcus aureus*
MRSA: Methicillin-resistant *Staphylococcus aureus*
HA-MRSA: Healthcare-associated MRSA
CA-MRSA: Community-associated MRSA
MSSA: Methicillin-sensible *Staphylococcus aureus*
CC: Clonal complex
ST: Sequence type
WHO: World Health Organization
MLST: Multiple Locus Sequence Typing
PFGE: Pulsed-Field Gel Electrophoresis
NGS: Next Generation Sequencing
