# Supplementary material for: High prevalence of t895 and t9364 spa types of methicillin-resistant Staphylococcus aureus in a tertiary-care hospital in Mexico: different lineages of clonal complex 5
Source: BMC Microbiol. 2020 Jul 20;20:213. doi: 10.1186/s12866-020-01881-w (PMC7370520; doi:10.1186/s12866-020-01881-w)
Supplement: Supplementary file 1 — Additional file 1: Table S1. Demographic and clinical characteristics of patients with Staphylococcus aureus infections who died or survived. Table S1 shows the demographic and clinical characteristics of patients with Staphylococcus aureus infections who died or survived. [file 12866_2020_1881_MOESM1_ESM.pdf]

Table S1. Demographic and clinical characteristics of patients with *Staphylococcus aureus* infections who died or survived

**Characteristic**	**Died (n=22)**	**Survived (n=169)**	**P value***
**Sex**			
Male (%)	13(59)	101(59.7)	0.95*
Female (%)	9(40.9)	68(40.2)	
**Age, years (mean, SD)**	46.3 (23.9)	35.2 (22.6)	0.034**
**Length of stay, days (Median, range)**	14.5 (1-105)	11 (1-90)	0.09***
**Underlying disease**			
Diabetes mellitus (%)	6 (27.3)	45 (26.6)	0.95*
Hypertension (%)	8 (36.4)	37 (21.9)	0.13*
Renal disease (%)	2 (9.1)	19 (11.2)	0.99****
Neoplasms (%)	0 (0)	10 (5.9)	0.57****
**Surgical procedures (%)**	8 (36.4)	76 (45)	0.44*
**Prior hospitalization (%)**	15 (68.2)	120 (71)	0.78*

*P value was calculated with chi-squared test, unless otherwise noted
**Student´s t test.
***Mann-Whitney U test.
****Fisher´s exact test
